# Antidepressant Effect of Enzymatic Porcine Placenta Hydrolysate in Repeated Immobilization Stress-Induced Ovariectomized Female Mice

**DOI:** 10.3390/cimb46060366

**Published:** 2024-06-17

**Authors:** Minsook Ye, Sharon Nguyen, Min Ju Kim, Jee Sun Hwang, Gun Won Bae, Keun-Hang Susan Yang, Insop Shim

**Affiliations:** 1 Department of Physiology, College of Medicine, Kyung Hee University, Seoul 02447, Republic of Korea; 2 Biological Sciences Program, Schmid College of Science and Technology, Chapman University, Orange, CA 92866, USA; 3 Department of R&D, Unimed Pharmaceuticals Inc., Unimed Bldg., Seoul 05567, Republic of Koreajeesun113@unimed.co.kr (J.S.H.); gwbae33@unimed.co.kr (G.W.B.); 4 Institute for Earth, Computing, Human and Observing (ECHO), Chapman University, Orange, CA 92866, USA

**Keywords:** depression, menopause, enzymatic porcine placenta hydrolysate (EPPH), neuroinflammation, RAW 264.7 cell

## Abstract

When postmenopausal women are under stress conditions, this exacerbates mood disorders and issues with neuroimmune systems. The porcine placenta is known to relieve menopausal depression in clinical trials, but its underlying mechanisms for depression and anti-inflammatory functions remain poorly defined. The present study was designed to examine the anti-inflammatory effects of enzymatic porcine placenta hydrolysate (EPPH) on LPS-induced levels of nitric oxide (NO), prostaglandin E2 (PGE2), corticosterone (CORT), and pro-inflammatory cytokine interleukin-1 beta (IL-1β) in RAW 264.7 macrophage cells. In addition, the neurite outgrowth of PC12 cells was evaluated to examine the effects of EPPH on neurite growth. To mimic the symptoms of women with menopause-related depression, a stressed ovariectomized (OVX) female mouse model was used to evaluate the antidepressant effects of EPPH. The female mice were randomly divided into five groups: (1) the sham-operated (Sham) group, (2) the OVX + repeated stress + saline-treated (OVX + ST) group, (3) the OVX + repeated stress + estradiol (0.2 mg/kg)-treated (positive control) group, (4) the OVX + repeated stress + EPPH (300 mg/kg)-treated (300) group, and (5) the OVX + repeated stress + EPPH (1500 mg/kg)-treated (1500) group. Female mice were OVX and repeatedly immobilization-stressed for 2 weeks (2 h/day). A tail suspension test was conducted on the 13th day, followed by the forced swimming test on the 14th day to assess the antidepressant effects of EPPH. After the behavioral tests, the levels of CORT, PGE2, and IL-1β were evaluated. In addition, c-Fos expression in the paraventricular nucleus (PVN) was evaluated using immunohistochemistry. The concentrations of NO, PGE2, and IL-1β stimulated by LPS were significantly reduced via the addition of EPPH to RAW 264.7 cells. EPPH significantly promoted neurite outgrowth in PC12 cells compared to that of the controls. In the tail suspension test, the duration of immobility was reduced in mice treated with EPPH 1500 compared to the OVX + ST group. The EPPH 1500 group had significantly decreased levels of c-Fos-positive neurons in the PVN and reduced levels of CORT and IL-1β in the serum of the Sham group. These results suggested that the high dose of EPPH administration induced the antidepressant-like effect in the ovariectomized mice with repeated stress via downregulating the levels of CORT, IL-1β, and PGE2 in the serum through reducing the expression of c-Fos in the PVN regions.

## 1. Introduction

Menopause is defined as the cessation of menstruation due to ovarian failure. In addition to physical symptoms, menopause is associated with a wide range of psychological symptoms including anxiety, depression, and cognitive impairment [1]. The onset of depression is correlated with a decrease in ovarian function [2,3]. Although estrogen therapy manages menopausal symptoms and improves related anxiety, depression, and memory impairment [4], a large body of evidence suggests that long-term estrogen therapy increases the risk of breast and ovarian cancer, stroke, and cardiovascular disease [5]. Most frequently, ovariectomized (OVX) animal models are used to mimic menopausal individuals because the lack of female hormones imitates estrogen deficiency in menopausal women [6]. OVX animal groups have evolved a credible tendency towards depressive-like behaviors due to estrogen insufficiency [7]. Previous studies have reported that chronic restraint stress-exposed OVX rodents show higher immobility time compared with non-stressed OVX rodents in tail suspension test (TST) and forced swimming test (FST) [7,8]. The onset of depression in groups has raised several questions regarding the mechanisms involved in brain pathology; however, the mechanical changes remain ambiguous.

In the pathogenesis of depression, immune responses to stressful stimuli within the central nervous system (CNS) are marked by the rapid activation of microglial cells and astrocytes, leading to the release of pro-inflammatory cytokines such as interleukin-1β (IL-1β), tumor necrosis factor (TNF)-α, and prostaglandin E2 (PGE2) [9]. Also, our previous study proved that the combination of ovariectomy and stress increases neuroinflammatory responses [10].

The activation of macrophages is a general feature of the early stages of pathogen infection. Lipopolysaccharide (LPS) is an important activator that binds to macrophages and produces an inflammatory response. RAW264.7 macrophage cell line has been developed as a primary experimental macrophage model for the study of macrophage signaling pathways and the research of macrophage-dependent inflammations due to ease of cell propagation, high efficiency for DNA transfection, sensitivity to RNA interference, and possession of receptors for many relevant ligands. During an inflammatory depressive response, pro-inflammatory mediators such as prostaglandin E2 (PGE2) are increased in the cerebrospinal fluid [11] and T-lymphocytes [12]. Similarly, during inflammatory processes, the release of nitric oxide (NO) and pro-inflammatory cytokine interleukin-1 beta (IL-1β) are modulated by the LPS-induced HPA response [13]. Overall, PGE2 and NO are induced by cyclooxygenase (COX-2) and nitric oxide synthase (iNOS) enzymes, respectively. The LPS-stimulated RAW264.7 cell inflammatory model is widely used as a model for studying the anti-inflammatory properties of drugs; therefore, understanding how EPPH treatment can impact these cells proves essential.

PC12 cells serve as a valuable model for studying neuronal differentiation, signaling, and various neurobiochemical/neurobiological events in the field of neuroscience [14]. Neurite extension has received a lot of attention as a biomarker of neurodevelopment and regeneration in vitro. The development of axonal and dendritic processes is a defining property of neuronal cell morphology and a decisive factor of neuronal cell interlinkability and function [15]. Hippocampal shrinkage has been connected to reduced neurotrophic backing and increased cortisol levels in patients with depression. In addition, the antidepressant fluoxetine has been shown to enhance neurite outgrowth and modulate the neurotrophic factor expression [16].

Women in transition to menopause boosts are more susceptible to stress and have a higher risk of developing mental disorders. With a disturbance to homeostasis, stress provokes the energization of the hypothalamic–pituitary–adrenal (HPA) axis. Initiated by the HPA axis, corticosterone (CORT) is released in the cortex of the adrenal gland and plays an adjustment role in stress in animals. CORT is also closely associated with depression. Treatment with CORT has been used to demonstrate major depression models in vitro and in vivo [17]. Thus, drugs that can reverse CORT-induced neurotoxicity may have therapeutic potential for the prevention or curing of depression [18].

In general, c-*fos*, one of the immediate early genes, is responsible for cell proliferation and differentiation following exposure to various extracellular stimuli. In addition, the expression of c-*fos* corresponds to the activation of specific circuitry of the brain related to the perception and integration of primary stimuli, as well as to neuroendocrine, autonomic, and behavioral responses. Immobilization stress can induce an intense expression of c-*fos* in several brain regions. In previous stress rat model studies, an increase in c-Fos protein was obseved in the paraventricular nucleus (PVN) of the hypothalamus [19]. Therefore, the expression of c-Fos protein can be used as a marker of neuronal activity and neuronal networks within multiple sites [20].

The placenta is a reservoir of bioactive molecules including hormones, proteins, and amino acids. One important bioactive includes the porcine placenta extract which is known for its anti-oxidant [21] or immune activity-enhancing effect [22]. In particular, bioactive compounds of human placental extract (HPE) are widely utilized to relieve menopausal symptoms [23]. In addition, the porcine placenta regulates malnutrition-induced fatigue by decreasing fatigue-related factors [24]. Previous studies have reported that the anti-inflammatory property of human placental extract ameliorates chronic inflammation and fatigue syndrome [23]. Additionally, stress models have suggested that human placental extract has therapeutic effects in decreasing stress-induced immobilization [25]. Nevertheless, the antidepressant effects of EPPH in an animal model of menopausal depression have not been reported yet.

In this study, we evaluated the effect of EPPH on relieving menopausal depressive symptoms. Specifically, the depressive-like behaviors were measured with a tail suspension test (TST) and a forced swimming test (FST). Moreover, the anti-inflammatory effects of EPPH on LPS-induced levels of NO, PGE2, CORT, and IL-1β in RAW 264.7 macrophage cells were analyzed in order to elucidate the mechanism of antidepressive actions via EPPH. Finally, neurite outgrowth in PC12 cells was evaluated to examine the effects of the EPPH on neurodevelopment/regeneration.

## 2. Material and Methods

### 2.1. Extracts from EPPH

EPPH was prepared using Ubio (Gangneung, Republic of Korea) as follows. The porcine placenta was washed with water three times to remove its blood and debris. The porcine placenta was then crushed and hydrolyzed with a proteolytic enzyme. After inactivating the protease via heating, it was filtered after ethanol precipitation and evaporated. After additional filtration, EPPH was autoclaved. EPPH was mainly composed of amino acids and peptides of mass 2–5 kD. The amino acid contents in EPPH were assessed using an amino acid analyzer (S433; SYKAM GmbH, Eresing, Germany). Peptide contents were calculated using the following equation: (total nitrogen amounts − total amino acids amounts)/total nitrogen amounts.

### 2.2. Cell Culture

#### 2.2.1. RAW 264.7 Cell

The macrophage RAW 264.7 cell line was purchased from the American Type Culture Collection (ATCC, Manassas, VA, USA) and maintained in Dulbecco’s modified minimum essential medium (DMEM) supplemented with 10% FBS, 100 μg/L of streptomycin, and 100 IU/mL of penicillin at 37 °C in a 5% CO_2_ atmosphere (HERAcell 150, Thermo Electron Corp., Waltham, MA, USA).

#### 2.2.2. PC12 Cell

The PC12 cell line was purchased from the American Type Culture Collection (ATCC, Manassas, VA, USA). The cells were grown DMEM-supplemented with 5% (*v*/*v*) heat-inactivated horse serum and 5% (*v*/*v*) heat-inactivated fetal bovine serum under 5% CO_2_/95% humidified air at 37 °C and fed on alternative days. The cells were sub-cultured every 3–4 days and the medium was changed every 2–3 days.

#### 2.2.3. MTT Cell Viability Assay

The 3-(4,5-dimethylthiazol-2-yl)-2,5-diphenyltetrazolium bromide (MTT) assay is a colorimetric assay for assessing cell viability and proliferation based on metabolic activity. Cell viability was determined using water-soluble tetrazolium salt (WST) assays and an EZ-Cytox cell viability assay kit (Daeil Lab Service Co., Ltd., Seoul, Republic of Korea). Briefly, RAW264.7 cells were preincubated overnight at a density of 1 × 10^4^ cells per well in 96-well plates, followed by treatment with various concentrations up to 100 μg/mL of EPPH. Twenty-four hours later, 10 μL of kit reagent was added to each well. After 1 h, the plates were read at 450 nm wavelength using a microplate reader (Molecular Devices, Sunnyvale, CA, USA). The results are expressed as a percentage of the untreated control. The cell viability assay was performed 3 times, and each assay was undertaken in triplicate.

### 2.3. Concentration of NO, PGE2, and IL-1β

#### 2.3.1. Inhibition of Nitric Oxide (NO) Production

The presence of nitrite was determined in the cell culture media and serum using a commercial NO detection kit (Abcam, Waltham, MA, USA). Protocols supplied with assay kits were used for the assay. Briefly, 100 μL of the cell culture medium with an equal volume of Griess reagent in a 96-well plate was incubated at room temperature for 10 min. Absorbance was measured at 540 nm using a microplate reader (Molecular Devices, CA, USA).

#### 2.3.2. Measurement of PGE2 and IL-1β Levels

RAW 264.7 cells were plated at 2 × 10^5^ cells/well in 6-well plates for PGE2 and IL-1β assays. The cells were then incubated with LPS (1 μg/mL) and various concentrations of EPPH for 24 h. The amounts of PGE2 (KGE004B, R&D Systems, Minneapolis, MN, USA) and IL-1β (ab197742, Abcam, MA, USA,) in cell culture supernatants were measured using ELISA kits according to the manufacturer’s instructions.

### 2.4. Evaluation of Neurite Outgrowth

The PC12 cells were plated onto a 6-well plate (35 mm plastic culture dishes), coated with poly-L-lysine (Sigma, Marlborough, MA, USA) at a density of 5 × 10^4^ cells/well, and cultured for 24 h at 37 °C in 5% CO_2_. After 24 h of culture, the culture medium was replaced with serum-free DF (DMEM/Ham’s F12, 1:1) and supplemented with transferrin, insulin, and progesterone. The cells were treated with different concentrations of EPPH diluted in sterile phosphate-buffered saline (PBS) at pH 7.4 to attain the desired EPPH concentrations of 0.1, 1, 10, 50, and 100 mg/mL. EPPH was not added to the control flasks. At least four to six flasks were prepared for every set of cell cultures. After culturing for 72 h in 5% CO_2_ at 37 °C, the cells were washed with PBS, 1 mL of 0.1% glutaraldehyde solution was added, then the cells were incubated for 30 min at room temperature. The length of the longest neurite of the individual cells was measured using an image processor system (Molecular Devices, CA, USA) that was attached to a phase-contrast microscope (200× magnification), using Imaging Software (Qwin Plus 271, Leica Microsystem imaging solutions Co., Ltd., Cambridge, UK).

### 2.5. Animals and Treatment

Seven-week-old C57/BL6 female mice from Samtako Animal Co. (Seoul, Republic of Korea) were used for this experiment. Although menopause is closely related to aging, surgically operating on seven-week-old female mice prior to the onset of reproductive senescence allows for the control of particular hormones without the effect of age as a confounding factor. The mice were kept under a regulated temperature (22–24 °C) with a 12 h light/dark cycle (lights on at 8:00 and lights off at 20:00). They were fed a standard diet and water until euthanasia. This experiment was approved by the Institutional Animal Care and Use Committee of Kyung Hee University (KHUAP(SE)-13-041).

Bilateral ovariectomy was conducted on all experimental animals except the sham-operated ones under intraperitoneal pentobarbital (50 mg/kg) anesthesia to eliminate endogenous ovarian steroid production. Ovariectomy involves the surgical removal of the fallopian tubes and ovaries through a midline incision. The sham operation was a fake surgical intervention through the midline incision through the skin and muscle.

**Figure 1:**
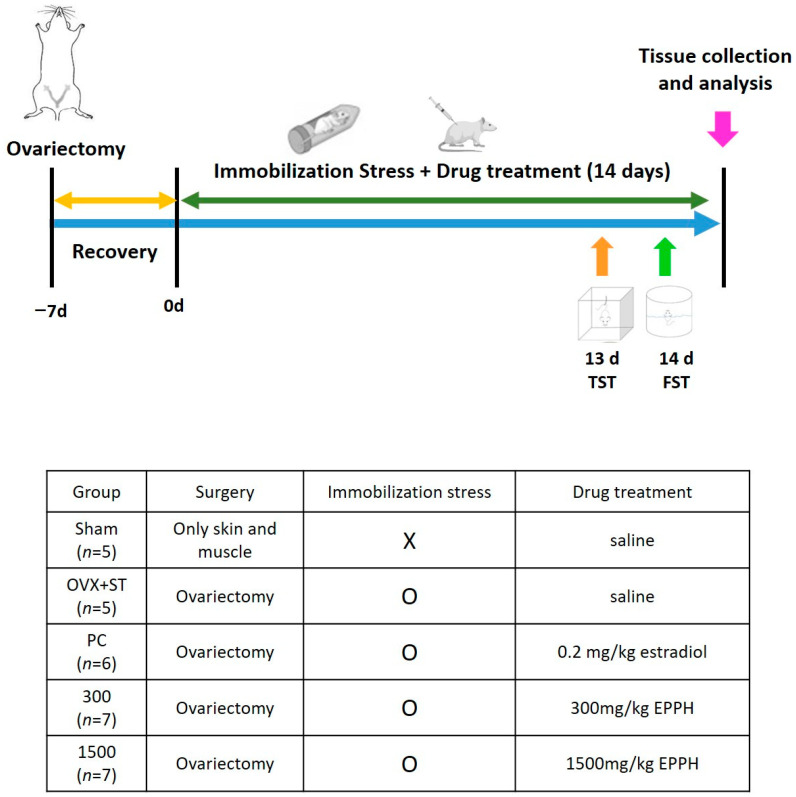
Animal groups and treatments in the experimental design of this study.

Mice were allowed to recover on a heated pad for up to 1 h following surgery and then returned to the home cage. Post-surgery, female mice were provided with up to 0.5 mL saline i.p. for rehydration. The site of operation was sterilized with Povidone Iodine Solution (Korea pharma Co., Hwaseong, Republic of Korea) and ethanol swabs three times. The exclusion criteria were either infection, death, or overt abnormal behavior of pain and distress. After 7 days of postoperative recovery, the mice were forced into an immobilizer device (disposable rodent restraint cone) to be put under stress for 2 h (13:00–15:00) for 2 weeks. Sterile saline was orally administered to the Sham and OVX + ST groups, while the other groups were administered the appropriate doses of the extract once a day for 2 weeks. Drug administration began 30 min before the immobilization stress. The experimental schedule is shown in Figure 1. Randomization was carried out as follows: After 7 days of arrival, animals were pre-weighed. A total of 30 mice were split into 5 different weight groups (*n* = 5–7 per group). Each animal was assigned a temporary random number within its respective weight range group, minimizing potential bias of weight. Then, bilateral ovariectomy and the sham operation were performed. The animals were monitored for 1 week post-surgery. Postoperative recuperation was monitored for 1 week. Sham-operated mice were defined as the Sham group as the control (drug-naïve; the operated-only abdominal incision and non-stressed group, Sham, *n* = 5); the ovariectomized, stressed, and saline-treated mice were defined as the OVX + ST group (drug-naïve OVX, *n* = 5) (drug-naïve OVX, *n* = 5); the ovariectomized, stressed, and subcutaneously treated with estradiol 1 μg/kg group (PC, *n* = 6); the ovariectomized, stressed, and treated orally with (EPPH) 300 mg/kg group (EPPH 300, *n* = 7); and the stressed and treated orally with (EPPH) 1500 mg/kg group (EPPH 1500, *n* = 7).

Blinding was carried out as follows. For each mouse, three different investigators were involved as follows: A first experimenter administered the treatment based on the randomization table. This investigator was the only person aware of the treatment group allocation. A second experimenter performed the surgical and IMO procedures. A third experimenter (also unaware of treatment) assessed behavioral tests and the IHC and ELISA or analyzed the data.

### 2.6. Behavioral Tests

All behavioral testing and scoring in this study were performed by a skilled observer blind to the experimental conditions.

#### 2.6.1. Tail Suspension Test

After 13 days of immobilization, mice were subjected to a TST. The total duration of immobility induced by tail suspension was measured according to the method of Steru [26]. Briefly, a mouse was taped approximately 1 cm from the tip of its tail and suspended upside down with its head approximately 10 cm above the floor in each chamber. The total duration of immobility was measured over 5 min.

#### 2.6.2. Forced Swimming Test

After 2 weeks of immobilization, the mice were subjected to a FST. At room temperature, the cylinder was filled with water to a depth of 30 cm, and no mice could touch the bottom with their tail. Mice were subjected to pre-swimming for 5 min on the day prior to experimentation [27]. All mice were forced to swim for 6 min, and the duration of immobility was observed and measured during the final 5 min of the test. During forced swimming for 5 min, the animals showed behaviors such as climbing, swimming, and immobility. Immobility was calculated as the length of time that did not show any movement [27].

### 2.7. c-Fos-like Immunohistochemistry

After sacrificing the mice, the brains were dissected, then post-fixed in 4% formaldehyde overnight, and placed in 30% sucrose solution for 24 h at 4 °C. Samples were cut into 30 μm thickness and the sections were kept at −20 °C. The brain sections were washed in PBS three times for 10 min and then quenched for 10 min at RT 3% H2O2 in PBS. Samples were rinsed in PBS three times for 10 min and blocked for 1 h at RT 0.2% triton X-100 (Sigma) and 1.5% bovine serum albumin (BSA) (Sigma, MA, USA) in PBS. Sections were rinsed in PBS containing 0.5% BSA three times for 10 min. The sections were kept overnight with gentle shaking at RT with PBS containing 0.5% BSA and then primary antibodies: rabbit anti-c-Fos (c-Fos; 1:2000; sc-52, Santa Cruz Biotechnology, Santa Cruz, CA, USA). The sections and then were washed in PBS containing 0.5% BSA three times for 10 min and kept for 2 h at RT in biotin-conjugated rabbit secondary antibody (1:500, PK-6101, Vector Laboratories, Burlingame, CA, USA). Brains were washed in PBS containing 0.5% BSA three times for 10 min and kept in an avidin–biotin complex kit (Vectastain ABC kit; Vector Laboratories, Burlingame, CA, USA), and then in DAB for 3 min. Labeled tissue sections were then mounted on gelatin-coated slides and analyzed under a bright-field microscope (Olympus Scientific Solutions Americas Corp., Center Valley, PA, USA).

### 2.8. Corticosterone (CORT), IL-1β Assay, and PGE2

After testing, the animals were anesthetized. Blood was collected quickly via intracardiac puncture to determine corticosterone concentration. Collected serum from the blood was stored at −20 °C until its use. The concentrations of corticosterone (ADI-900-097, Enzo Life Sciences, New York, NY, USA), IL-1β (ab197742, Abcam, MA, USA), and PGE2 (KGE004B, R&D Systems, Minneapolis, MN, USA) in the serum were measured according to the manufacturer’s instructions.

### 2.9. Statistical Analysis

Statistical comparisons were performed for the behavioral and histochemical studies using the one-way analysis of variance (ANOVA), followed by Tukey’s post hoc test. *p*-values < 0.05 were considered to indicate statistically significant differences. To ascertain whether the distribution of the data was normal, the Shapiro–Wilk test was applied. All of the results are presented as means ± standard error of the mean. All statistical analyses were performed using SPSS 23.0 software (SPSS Inc., Chicago, IL, USA).

## 3. Results

### 3.1. Effects of EPPH on the Viability of RAW 264.7 Cells and PC12 Cells

**Figure 2:**
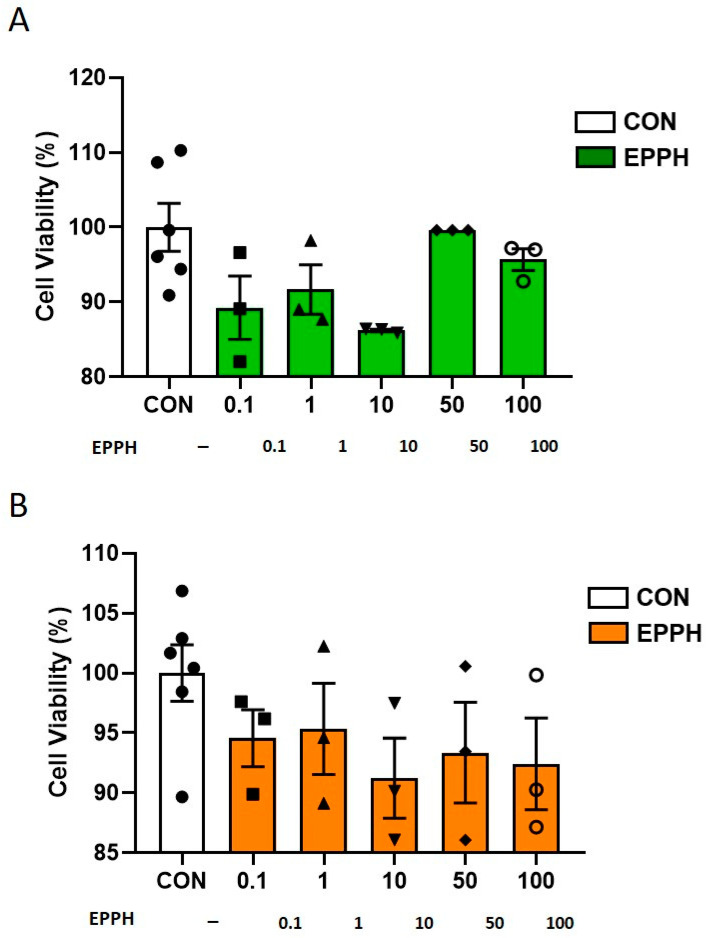
Effects of EPPH on cell viability in RAW 264.7 mouse macrophages and PC12 cells. After 24 h treatment, (**A**) RAW 264.7 cell and (**B**) PC12 cell viabilities were evaluated using a modified MTT assay. One-way ANOVA was followed by Tukey’s post hoc test.

Before analyzing the anti-inflammatory effects of EPPH, we examined the cytotoxic effects of EPPH on RAW 264.7 cells and PC12 cells via MTT assay. Treatment with EPPH (0.1, 1, 10, 50, and 100 μg/mL) did not appear cytotoxic to the RAW 264.7 cells (Figure 2A) and PC12 cells (Figure 2B). Hence, these results indicate that EPPH did not have any cytotoxic effects.

### 3.2. EPPH Inhibits LPS-Induced NO and PGE2 Production

**Figure 3:**
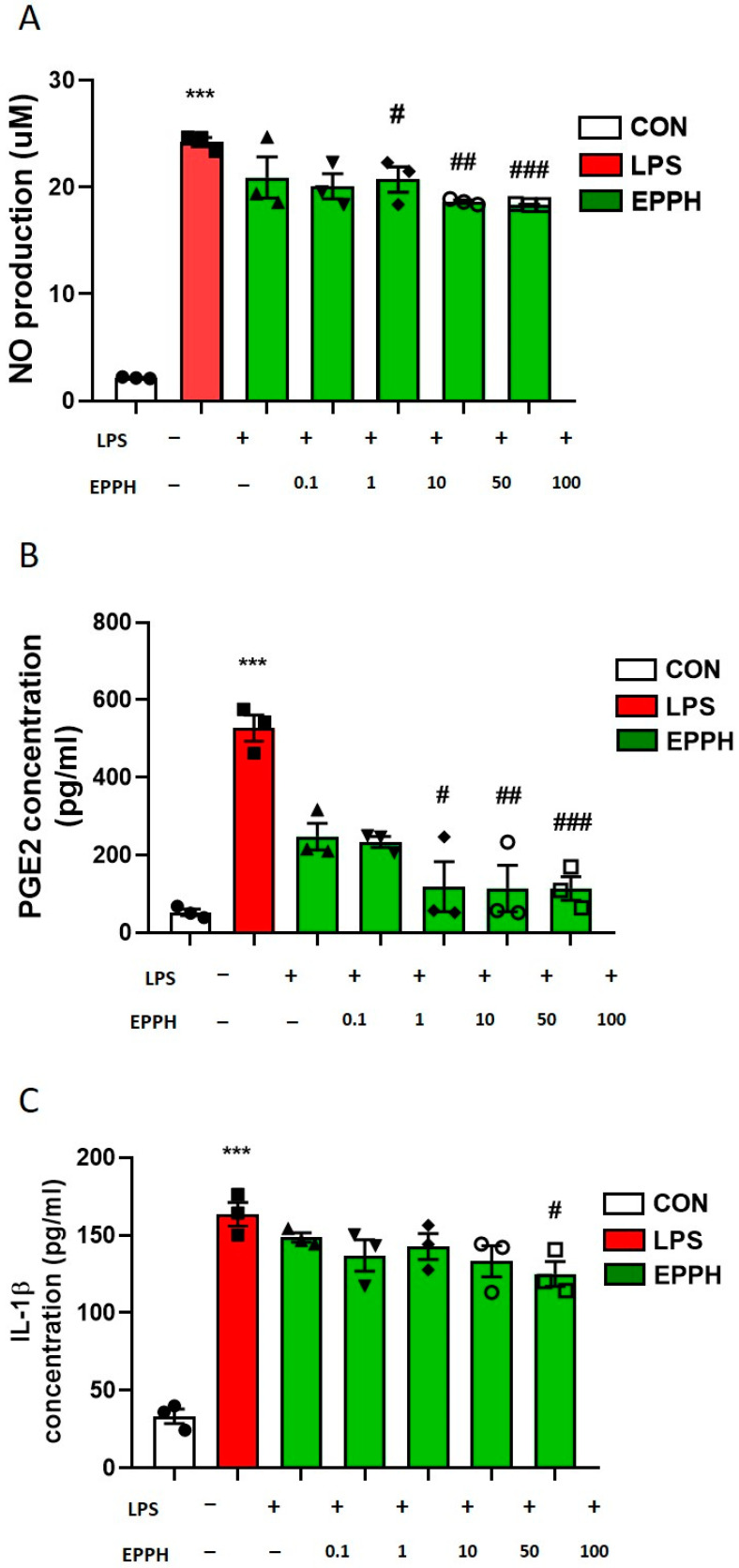
Effects of EPPH on the production of (**A**) NO, (**B**) PGE2, and (**C**) IL-1β in LPS-induced RAW 264.7 mouse macrophages. The fluorescence intensity of each cytokine in the culture medium was measured using a Multiplex bead-based cytokine assay after 24 h of treatment. Values are the means ± S.E.M. of three independent experiments (*n* = 3). One-way ANOVA was followed by Tukey’s post hoc test, *** *p* < 0.001 vs. Con; ^#^ *p* < 0.05, ^##^ *p* < 0.01, ^###^ *p* < 0.001 vs. LPS.

RAW264. 7 cells are widely used as a model for studying the anti-inflammatory properties of drugs; therefore, understanding how EPPH treatment can impact these cells proves essential. To evaluate the effects of EPPH on LPS-induced NO, PGE2, and IL-1β production in RAW 264.7 cells, cells were treated with/without EPPH (0.1, 1, 10, 50, or 100 μg/mL) for 1 h and then treated with LPS (1 μg/mL) for 24 h. Treatment with EPPH extract at concentrations of 10 (*p* < 0.05), 50 (*p* < 0.01), and 100 (*p* < 0.001) μg/mL achieved a statistically significant decrease in NO production in cells given a stimulus of LPS in a dose-dependent manner (Figure 3A). Levels of PGE2 production (Figure 3B) showed a statistically significant increase (*p* < 0.001) following the stimulation of cells with LPS. The treatment of RAW 264.7 cells with EPPH at concentrations of 10, 50, and 100 μg achieved a statistically significant reduction (*p* < 0.05, *p* < 0.01, and *p* < 0.001, respectively) in the LPS-induced production of PGE2 in a concentration-dependent manner (Figure 3B). We determined the NO and PGE2 levels in the medium and observed that EPPH decreased the production of NO and PGE2 in a dose-dependent manner. To better understand the inhibitory effects of EPPH extract on inflammation, the concentration of IL-1β was investigated via ELISA in RAW 264.7 cells stimulated by LPS. The expression of IL-1β at this concentration exhibited a statistically significant increase (*p* < 0.001) following the stimulation of the cells with LPS. Treatment with EPPH was associated with the inhibition of the LPS-induced concentration of IL-1β in RAW 264.7 cells (Figure 3C), indicating that EPPH inhibited the synthesis of pro-inflammatory cytokines in activated macrophages.

### 3.3. Effect of EPPH on Neurite Outgrowth in PC12 Cells

**Figure 4:**
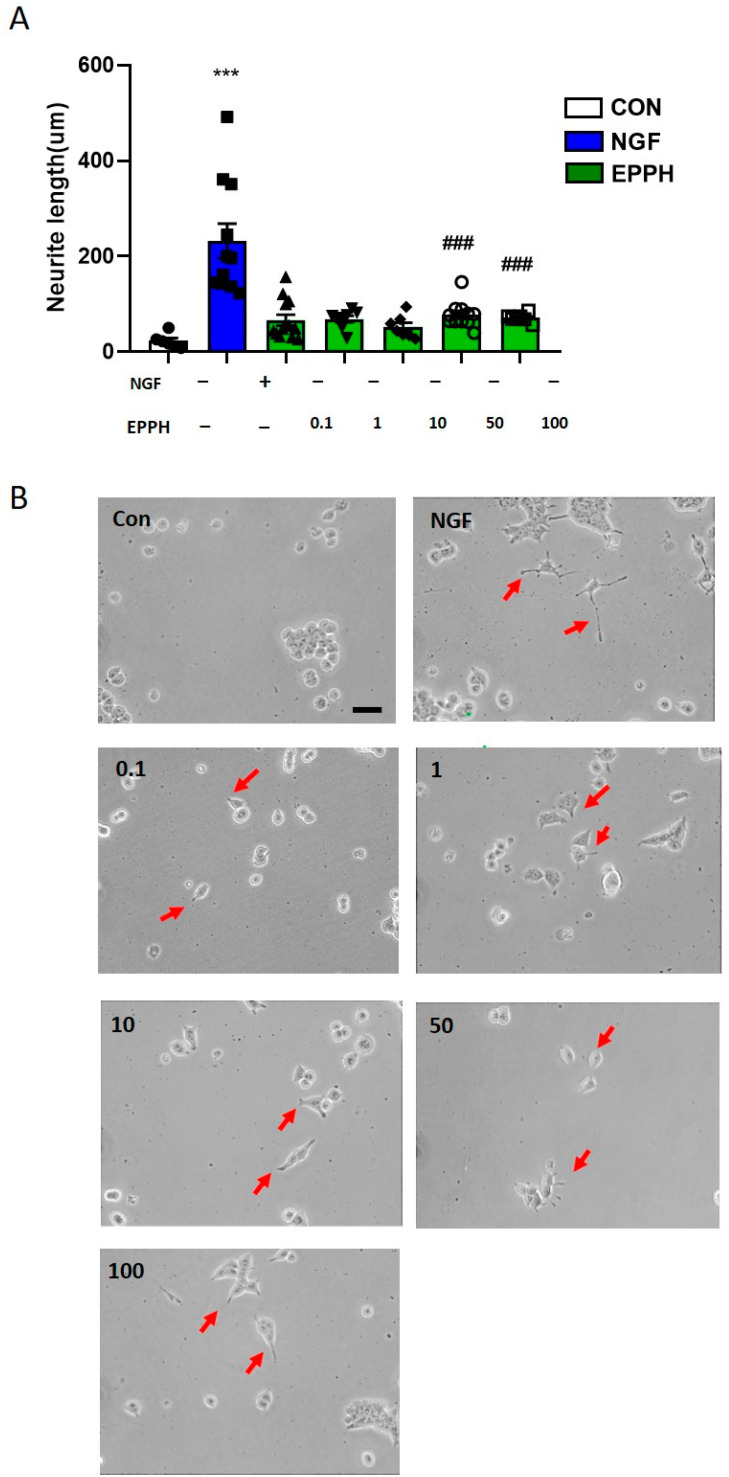
Effects of EPPH on neurite outgrowth in PC12 cells. (**A**) Morphological changes of PC12 cells treated with Con, NGF (20 ng/mL), and EPPH (0.1, 1, 10, 50, and 100 ng/mL) were visualized under light microphotography. (**B**) Neurite-bearing cells were quantitatively analyzed as described in the measurement of neurite outgrowth. Values are the means ± S.E.M. of three independent experiments (*n* = 3). The scale bar represents 100 μm. One-way ANOVA was followed by Tukey’s post hoc test, *** *p* < 0.001 vs. Con; ^###^ *p* < 0.001 vs. NGF. Red arrows represent neurite extensions.

The rat pheochromocytoma PC12 cells are commonly used to investigate neurite extension. To investigate the effect of EPPH on neurite outgrowth in PC12 cells, cells were treated with control (non-treatment), NGF (100 ng/mL), or EPPH (0.1–100 µg/mL). As shown in Figure 4A, EPPH treatment promoted neurite extension in PC12 cells. NGF treatment (100 ng/mL) also significantly increased neurite outgrowth (*p* < 0.001). Figure 4B shows the morphology of PC12 cells with neurite outgrowth after 72 h of treatment with NGF (100 ng/mL), EPPH (50 and 100 μg/mL), and control.

### 3.4. Antidepressant-like Effect of EPPH

**Figure 5:**
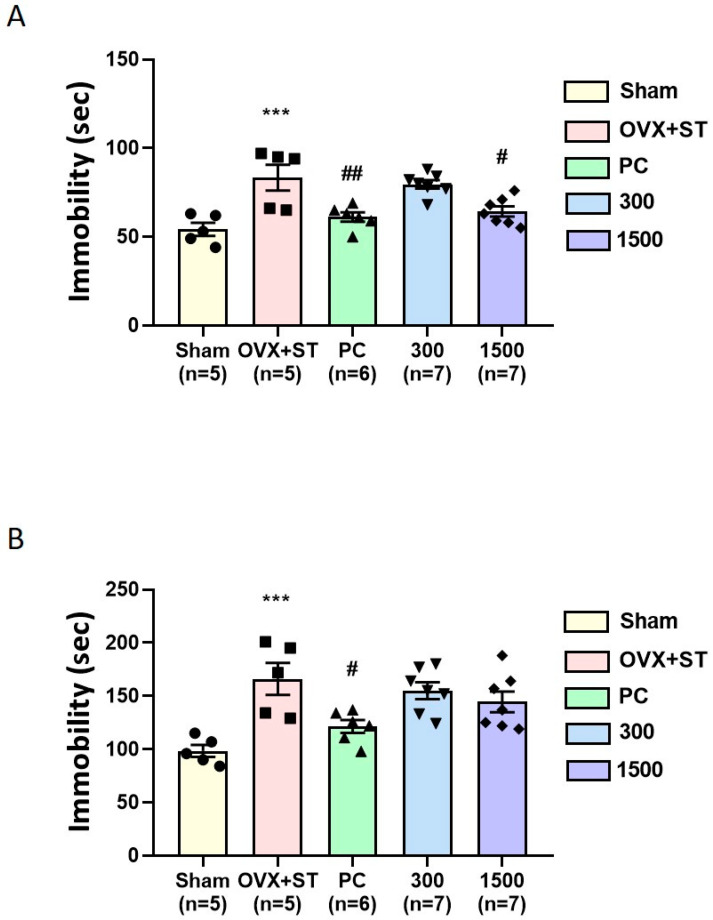
Effects of EPPH to reduce immobility time on TST (**A**) and FST (**B**) in mice. Mice received saline, EPPH (300 and 1500 mg/kg), or estradiol (0.2 mg/kg). Each value is represented as means ± S.E.M. Statistical analysis was performed using one-way ANOVA followed by Tukey’s post hoc test. One-way ANOVA was followed by Tukey’s post hoc test, *** *p* < 0.001 vs. Sham group; ^#^ *p* < 0.05, ^##^ *p* < 0.01 vs. OVX + ST group, respectively.

The antidepressant-like activity of EPPH was assessed in OVX mice subjected to immobilization stress by measuring the duration of immobility during FSTs and TSTs (Figure 5). Figure 5A shows the effect of EPPH on the duration of immobility during the TST. The Sham group showed a decrease in immobility time compared with the OVX + ST group (*p* < 0.001). The PC group produced a significant reduction in immobility duration compared with the OVX + ST group (*p* < 0.01). In addition, EPPH (1500 mg/kg) significantly decreased the immobility time duration compared with the OVX + ST group (*p* < 0.05). Figure 5B shows the effect of EPPH on the duration of immobility during the FST. The Sham group showed a significant reduction in immobility compared with the OVX + ST group (*p* < 0.001). The PC group produced a significant reduction in immobility duration in the FST (*p* < 0.05). However, EPPH (300 and 1500 mg/kg) had no effect on the FST.

### 3.5. Effects of EPPH on c-Fos Expression in the PVN

**Figure 6:**
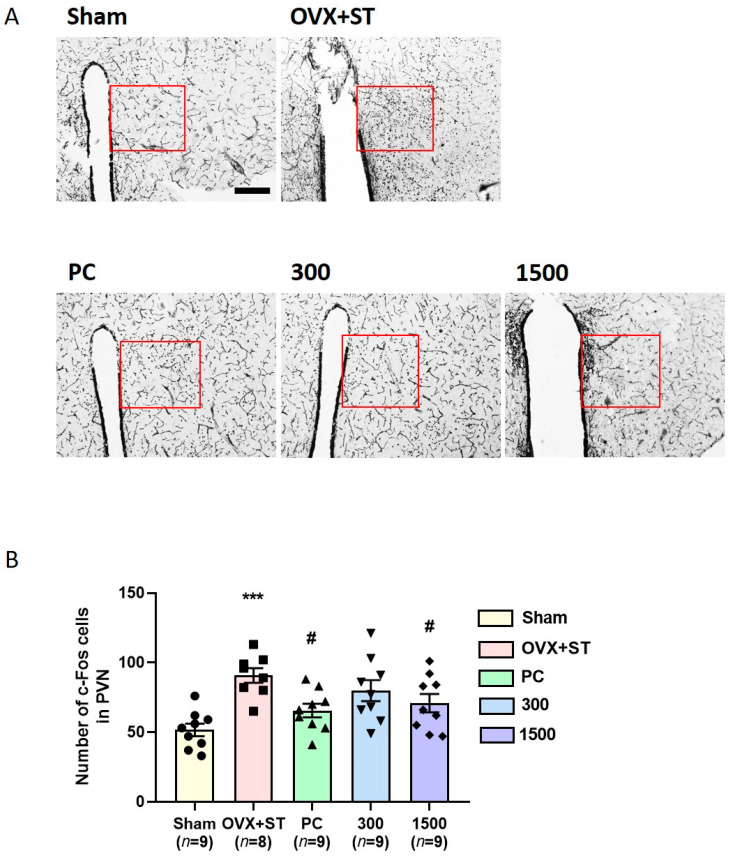
Effect of EPPH on the expression of c-Fos-positive cells in the PVN region. (**A**) Representative photographs of c-Fos-positive cells in the PVN region. (**B**) Results of c-Fos reactivity, counted in red box, were analyzed by performing separate one-way ANOVA of cells among the groups. Each value represents the mean  ±  S.E.M. (*n* = 9, respectively). The scale bar represents 50 μm. One-way ANOVA was followed by Tukey’s post hoc test, *** *p* < 0.001 vs. Sham group; ^#^ *p* < 0.05 vs. OVX + ST group. Red boxes represent the PVN region.

The results of c-Fos expression in the paraventricular area of mice are shown in Figure 6. The OVX + ST group showed a significant increment in the number of c-Fos neurons compared with the Sham group (*p* < 0.001). The PC group and EPPH (1500 mg/kg) group showed a significant reduction in c-Fos-positive neurons compared with the OVX + ST group (*p* < 0.05).

### 3.6. Effect of EPPH on Immobilization-Induced Change of Corticosterone, PGE2, and IL-1β Concentrations in the Serum

**Figure 7:**
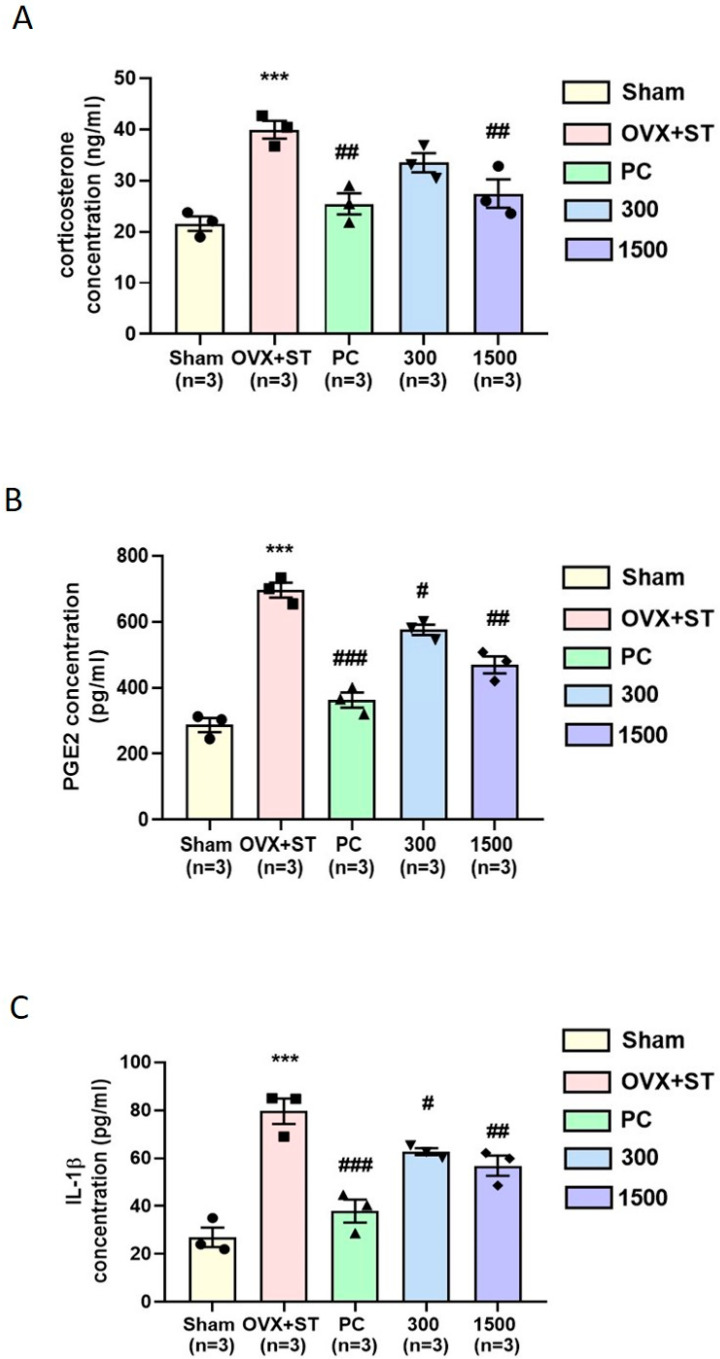
Effect of EPPH on immobilization-induced change of (**A**) CORT, (**B**) PGE2, and (**C**) IL-1β concentrations in the serum. The data were expressed as means ± S.E.M. (*n* = 3, respectively). Statistical analysis was conducted using a one-way analysis of variance, followed by Tukey’s post hoc test where appropriate, *** *p* < 0.001 vs. Sham group; ^#^ *p* < 0.05, ^##^ *p* < 0.01, ^###^ *p* < 0.001 vs. OVX + ST group, respectively.

CORT levels in Sham-, OVX + ST-, PC-, and EPPH-treated mice were measured. As shown in Figure 7A, the Sham group showed a significant decrease in CORT levels compared to the OVX + ST group (*p* < 0.001). The PC-and EPPH 1500-treated groups showed a significant decrease in CORT levels compared to the OVX + ST group (*p* < 0.01). The serum concentrations of PGE2 of the Sham-, OVX + ST-, PC-, and EPPH-treated groups are shown in Figure 7B, the Sham group showed a significant decrease in PGE2 levels compared to the OVX + ST group (*p* < 0.001). The PC-, EPPH 300-, and EPPH 1500-treated mice showed significantly lower PGE2 serum levels than the OVX + ST-treated mice (*p* < 0.001, *p* < 0.05, and *p* < 0.01, respectively). The concentration of serum IL-1β in the Sham-, OVX + ST-, PC-, and EPPH-treated mice was measured. As shown in Figure 7C, the Sham group showed a significant decrease in IL-1β levels compared to the OVX + ST group (*p* < 0.001). The PC-, EPPH 300-, and EPPH 1500-treated groups exhibited a significant decrease in IL-1β levels compared to the OVX + ST group (*p* < 0.001, *p* < 0.05, and *p* < 0.01, respectively). Repeated stress increased the serum levels of CORT, IL-1β, and PGE2 in OVX + ST mice, but treatment with either EPPH or PC reduced the levels of all pro-inflammatory cytokines (Figure 7).

## 4. Discussion

During the transition to menopause, women experience drastic hormonal changes, resulting in a higher risk of depression. Currently, great concern surrounds estrogen replacement therapy’s potential intense side effects, such as thrombotic events, breast cancer, and dementia [28]. With these alarming insights in mind, the urgency is to investigate the underlying mechanisms of menopause-related depression and to seek a safer treatment approach. In this study, we found that EPPH attenuated the inflammatory response by regulating the expression and secretion of LPS-induced inflammatory mediators in vitro. Moreover, we demonstrated that the administration of EPPH reduced depressive-like behaviors and pro-inflammatory cytokine expression in vivo. The results showed that treatment with EPPH was associated with a decreased immobility time in the TSTs and reduced levels of CORT and IL-1β in the serum. Additionally, the administration of EPPH decreased the brain expression of c-Fos in the PVN, implying decreased stress levels. EPPH also stimulated transcription and neurite outgrowth in PC12 cells and decreased the NO and PGE2 production in RAW 264.7 cells. These results suggest that the administration of EPPH yielded antidepressant effects through pro-inflammatory cytokines in this menopausal animal model.

The pheochromocytoma PC12 cell line is widely used as a model to study various neuronal functions. PC12 cells respond to neurotrophic factors, including NGF and fibroblast growth factor (FGF), by differentiating into sympathetic neuron-like phenotypes characterized by neurite outgrowth and the expression of many neuron-specific proteins [29,30]. In this study, treatment with EPPH significantly increased neurite outgrowth in PC12 cells in a dose-dependent manner. This finding supports the hypothesis that EPPH improves the neurotrophic system.

Since NO and PGE2 are major inflammatory mediators, abnormal production will generate inflammation. Macrophages activated by LPS are known to produce inflammatory mediators such as NO and PGE2, in addition to numerous cytokines such as tumor necrosis factor-α (TNF-α), IL-1β, and IL-6 [4,5,6]. In the present study, we showed that in LPS-stimulated macrophages, EPPH dose-dependently inhibited NO and PGE2 production. The level of the inflammatory cytokine IL-1β was also downregulated by EPPH, suggesting that this was the mechanism underlying the observed suppression of NO and PGE2 production.

In menopausal research, animals that are OVX develop a predisposition to depressive-like behavior due to the lack of estrogen and physiological stress. In the FSTs and TSTs, ovariectomized mice were administered EPPH to examine its antidepressant effects. Moreover, the stress models were purposed to measure changes in depressive-like behaviors via total immobility time—increased immobility time reflected a behavioral despair [31,32]. A reduction in the duration of immobility indicates an antidepressant effect of the treatment drugs. In the current study, the OVX + ST group showed a significant increase in immobility time compared with the Sham group. More importantly, the EPPH 1500 and PC groups showed decreased immobility time in the TST, resulting in neurotransmitter modulation in different brain areas. In contrast, EPPH 300 and 1500 had no effect on the immobility time in the FSTs. It has been well established that estradiol treatment exerts an antidepressant-like effect in ovariectomized mice in depression despair animal tests while having no effect on sham-operated animals [33]. Therefore, it is possible that estradiol and EPPH treatment induce a decrease in immobility time in FSTs and TSTs compared to the OVX + ST treatment. Even though EPPH treatment produced an antidepressant-like effect in ovariectomized mice, we cannot exclude the possibility that EPPH treatment itself has an antidepressant-like effect on normal conditions. This should be examined in further studies.

Both the forced swimming test (FST) and the tail suspension test (TST) are the most commonly used behavioral tests to assess depressive-like behavior or to screen potential antidepressant drugs. While they share similarities in their aims and applications and are conceptually similar to each other, they differ in their methodologies and the behaviors they measure. Therefore, they do not show identical sensitivities to pharmacologic agents or to strain differences. Generally, the TST is considered more reliable and has greater sensitivity to antidepressants because the procedures of the two tests differ. In the FST, the degree of immobility in animals may be compounded by the shock of being dropped into water and/or exposed to pharmacologic agents [34]. Consistent with the previous findings [8], it was shown that treatment with estrogen, used as a positive control, reduced immobility time in both tests, with a more pronounced effect observed in the TST than in the FST, suggesting that the TST is more sensitive and reliable and considered to have good predictive validity. However, it should be noted that while a reduction in immobility treated with EPPH in the FST did not reach statistical significance, its trend is similar to that observed in the TST.

Evidence suggests that exposure to chronic stress markedly elevates serum CORT levels and induces depressive-like behavior in mice. In the present study, OVX + ST mice showed significantly higher serum corticosterone levels than the Sham group, suggesting that the HPA axis was activated in OVX animals. Furthermore, our results showed that ovariectomy-induced elevations in serum corticosterone levels were accompanied by depression-like behavior, which is similar to stress-induced endocrine responses and behavioral changes. Overall, our study demonstrated that the administration of EPPH reduced CORT levels in the serum of OVX + ST mice.

The placenta is a temporary organ that conveys oxygen and nutrients to the fetus during pregnancy [35]. Placental extracts contain a variety of bioactive molecules such as proteins, peptides, estrogen, glycosaminoglycans, amino acids, RNA, and DNA. The combination of bioactive compounds alludes to regenerative effects, such as skincare [36], fatigue reduction [37], and relief from menopausal symptoms [23]. Recently, porcine placental extract has been developed as an oral supplement for the same purpose as human placental extract. The oral administration of FPP has been reported to be significantly effective in reducing knee pain and wrinkle width. A previous study suggested that the improvement of vaginal atrophy, a significant increase in uterine weight, and an increasing trend in estradiol were observed after oral PPE administration in castrated mice [23]. Hormone replacement therapy is taken to be the most effective treatment for menopausal syndrome, but various disadvantages and side effects have been reported, including the increased risk of breast and ovarian cancer and stroke [38]. Previous studies have suggested that the clinical and practical use of EPPH is assumed to be safe. EPPH is useful as an alternative to antidepressants without adverse effects. However, few reports are available on the efficacy and safety of placental extracts in menopausal animals.

EPPH contains a variety of small peptides and amino acids including arginine, isoleucine, and leucine, most of which having been demonstrated to possess significant anti-inflammatory and antidepressant properties [39,40,41]. It has been reported that decreased levels of leucine, valine, and isoleucine were associated with major depression [42], and arginine also has inflammatory properties [43]. Therefore, these major components of EPPH may have been responsible for the anti-inflammation and antidepressant effects shown in the present study. Future studies are needed to understand the precise neurochemical and behavioral effects of these components in the OVX mouse model.

This study has several limitations. First, we have tested representative markers, IL-1β, PGE2, and NO as pro-inflammatory mediators to investigate the anti-inflammatory effect of FPP in the present study. In further studies, other biomarkers such as TNFα, IL-6, or NF-κB are needed to examine the more precise mechanisms underlying EPPH actions. Second, we have selected the OVX model in young mice to study the effects of hormonal changes, particularly estrogen withdrawal, mimicking postmenopausal conditions in women. While this model has been commonly utilized in a variety of studies, it also has some weaknesses. OVX mice may not sufficiently represent the aging process since they primarily mimic the hormonal changes associated with menopause rather than aging-related factors. OVX also produces a relatively abrupt and complete loss of ovarian hormones, and this may differ from the gradual decline seen in the natural process of menopause. Taking this into consideration, combining the OVX with aging models can enhance the robustness of our findings in further studies.

In summary, the findings of the present study demonstrated that EPPH produced promising antidepressant effects evidenced by reduced immobility time in TST and FST via regulations of corticosterone and anti-inflammatory responses. As this study provides preliminary findings, the goal is to demonstrate a more sustainable alternative to treating depression with a combination treatment plan of holistic and pharmaceutical medicine.

## Data Availability

The data used to support the findings of this study are available from the corresponding author upon request.

## References

[1] Ali A.M., Ahmed A.H., Smail L. (2020). Psychological Climacteric Symptoms and Attitudes toward Menopause among Emirati Women. Int. J. Environ. Res. Public Health.

[2] Liang G., Kow A.S.F., Yusof R., Tham C.L., Ho Y.C., Lee M.T. (2024). Menopause-Associated Depression: Impact of Oxidative Stress and Neuroinflammation on the Central Nervous System-A Review. Biomedicines.

[3] Turek J., Gasior L. (2023). Estrogen fluctuations during the menopausal transition are a risk factor for depressive disorders. Pharmacol. Rep..

[4] Herson M., Kulkarni J. (2022). Hormonal Agents for the Treatment of Depression Associated with the Menopause. Drugs Aging.

[5] Kohn G.E., Rodriguez K.M., Hotaling J., Pastuszak A.W. (2019). The History of Estrogen Therapy. Sex. Med. Rev..

[6] Tao X., Yan M., Wang L., Zhou Y., Wang Z., Xia T., Liu X., Pan R., Chang Q. (2020). Effects of estrogen deprivation on memory and expression of related proteins in ovariectomized mice. Ann. Transl. Med..

[7] Ye M., Jang D., Kim J.S., Kim K., Shim I. (2019). Effects of Camellia Sinensis Extract on Repeated Restraint Stress-Induced Ovariectomized Female Rats. BioMed Res. Int..

[8] Shaif N.A., Chang D.H., Cho D., Kim S., Seo D.B., Shim I. (2018). The Antidepressant-Like Effect of Lactate in an Animal Model of Menopausal Depression. Biomedicines.

[9] Mandal S., Spoorthy M.S., Godi S.M., Nanda R., Mukherjee B., Mishra N.R. (2023). Inflammatory Markers in Patients With Major Depressive Disorder: A Prospective, Clinic-Based, Cohort Study From India. Cureus.

[10] Park H.J., Shim H.S., Shim I. (2020). The Differential Role of Cytokines on Stress Responses in a Menopause Rat Model. Front. Psychiatry.

[11] Kouba B.R., de Araujo Borba L., Borges de Souza P., Gil-Mohapel J., Rodrigues A.L.S. (2024). Role of Inflammatory Mechanisms in Major Depressive Disorder: From Etiology to Potential Pharmacological Targets. Cells.

[12] Wang H., He Y., Sun Z., Ren S., Liu M., Wang G., Yang J. (2022). Microglia in depression: An overview of microglia in the pathogenesis and treatment of depression. J. Neuroinflamm..

[13] Kwon H.S., Koh S.H. (2020). Neuroinflammation in neurodegenerative disorders: The roles of microglia and astrocytes. Transl. Neurodegener..

[14] Sampieri L., Funes Chaban M., Di Giusto P., Rozes-Salvador V., Alvarez C. (2021). CREB3L2 Modulates Nerve Growth Factor-Induced Cell Differentiation. Front. Mol. Neurosci..

[15] Shim T., Kim J.Y., Kim W., Lee Y.I., Cho B., Moon C. (2024). Cullin-RING E3 ubiquitin ligase 4 regulates neurite morphogenesis during neurodevelopment. iScience.

[16] Meng P., Zhu Q., Yang H., Liu D., Lin X., Liu J., Fan J., Liu X., Su W., Liu L. (2019). Leonurine promotes neurite outgrowth and neurotrophic activity by modulating the GR/SGK1 signaling pathway in cultured PC12 cells. Neuroreport.

[17] Bai G., Qiao Y., Lo P.C., Song L., Yang Y., Duan L., Wei S., Li M., Huang S., Zhang B. (2022). Anti-depressive effects of Jiao-Tai-Wan on CORT-induced depression in mice by inhibiting inflammation and microglia activation. J. Ethnopharmacol..

[18] Yu X.D., Zhang D., Xiao C.L., Zhou Y., Li X., Wang L., He Z., Reilly J., Xiao Z.Y., Shu X. (2022). P-Coumaric Acid Reverses Depression-Like Behavior and Memory Deficit Via Inhibiting AGE-RAGE-Mediated Neuroinflammation. Cells.

[19] Moench K.M., Breach M.R., Wellman C.L. (2019). Chronic stress produces enduring sex- and region-specific alterations in novel stress-induced c-Fos expression. Neurobiol. Stress..

[20] Cruz-Mendoza F., Jauregui-Huerta F., Aguilar-Delgadillo A., Garcia-Estrada J., Luquin S. (2022). Immediate Early Gene c-fos in the Brain: Focus on Glial Cells. Brain Sci..

[21] Nagae M., Nishio T., Ohnuki K., Shimizu K. (2022). Effects of oral administration of equine placental extract supplement on the facial skin of healthy adult women: A randomized, double-blind, placebo-controlled study. Health Sci. Rep..

[22] Ding J., Maxwell A., Adzibolosu N., Hu A., You Y., Liao A., Mor G. (2022). Mechanisms of immune regulation by the placenta: Role of type I interferon and interferon-stimulated genes signaling during pregnancy. Immunol. Rev..

[23] Lee J.Y., Lee C., Yoon S.H., Choi H. (2020). Effect of porcine placental extract on menopausal symptoms in postmenopausal women: A prospective, randomized, double-blind, placebo-controlled trial. Taiwan J. Obstet. Gynecol..

[24] Lee K.K., Choi W.S., Yum K.S., Song S.W., Ock S.M., Park S.B., Kim M.J. (2012). Efficacy and safety of human placental extract solution on fatigue: A double-blind, randomized, placebo-controlled study. Evid. Based Complement. Alternat Med..

[25] Park H.J., Shim H.S., Lee S., Hahm D.H., Lee H., Oh C.T., Han H.J., Ji H.J., Shim I. (2018). Anti-stress effects of human placenta extract: Possible involvement of the oxidative stress system in rats. BMC Complement. Altern. Med..

[26] Steru L., Chermat R., Thierry B., Simon P. (1985). The tail suspension test: A new method for screening antidepressants in mice. Psychopharmacology.

[27] Porsolt R.D., Bertin A., Jalfre M. (1977). Behavioral despair in mice: A primary screening test for antidepressants. Archives Internationales de Pharmacodynamie et de Therapie.

[28] Yuk J.S., Lee J.S., Park J.H. (2023). Menopausal hormone therapy and risk of dementia: Health insurance database in South Korea-based retrospective cohort study. Front. Aging Neurosci..

[29] Leonov G., Salikhova D., Shedenkova M., Bukharova T., Fatkhudinov T., Goldshtein D. (2023). Comparative Study of the Protective and Neurotrophic Effects of Neuronal and Glial Progenitor Cells-Derived Conditioned Media in a Model of Glutamate Toxicity In Vitro. Biomolecules.

[30] Xie D., Deng T., Zhai Z., Sun T., Xu Y. (2022). The cellular model for Alzheimer’s disease research: PC12 cells. Front. Mol. Neurosci..

[31] Qiao Y., Zhao J., Li C., Zhang M., Wei L., Zhang X., Kurskaya O., Bi H., Gao T. (2020). Effect of combined chronic predictable and unpredictable stress on depression-like symptoms in mice. Ann. Transl. Med..

[32] Yin R., Zhang K., Li Y., Tang Z., Zheng R., Ma Y., Chen Z., Lei N., Xiong L., Guo P. (2023). Lipopolysaccharide-induced depression-like model in mice: Meta-analysis and systematic evaluation. Front. Immunol..

[33] Tantipongpiradet A., Monthakantirat O., Vipatpakpaiboon O., Khampukdee C., Umehara K., Noguchi H., Fujiwara H., Matsumoto K., Sekeroglu N., Kijjoa A. (2019). Effects of Puerarin on the Ovariectomy-Induced Depressive-Like Behavior in ICR Mice and Its Possible Mechanism of Action. Molecules.

[34] Castagne V., Moser P., Roux S., Porsolt R.D. (2010). Rodent models of depression: Forced swim and tail suspension behavioral despair tests in rats and mice. Curr. Protoc. Neurosci..

[35] Burton G.J., Jauniaux E. (2023). The human placenta: New perspectives on its formation and function during early pregnancy. Proc. Biol. Sci..

[36] Nagae M., Nagata M., Teramoto M., Yamakawa M., Matsuki T., Ohnuki K., Shimizu K. (2020). Effect of Porcine Placenta Extract Supplement on Skin Condition in Healthy Adult Women: A Randomized, Double-Blind Placebo-Controlled Study. Nutrients.

[37] Yoon D.H., Han G.Y., Hwang S.S., Lee D.W., Kim J.S., Kim K., Kim J., Song W. (2020). The Effect of Fermented Porcine Placental Extract on Fatigue-Related Parameters in Healthy Adults: A Double-Blind, Randomized, Placebo-Controlled Trial. Nutrients.

[38] Mehta J., Kling J.M., Manson J.E. (2021). Risks, Benefits, and Treatment Modalities of Menopausal Hormone Therapy: Current Concepts. Front. Endocrinol..

[39] Fellendorf F.T., Platzer M., Pilz R., Rieger A., Kapfhammer H.P., Mangge H., Dalkner N., Zelzer S., Meinitzer A., Birner A. (2019). Branched-chain amino acids are associated with metabolic parameters in bipolar disorder. World J. Biol. Psychiatry.

[40] Baranyi A., Amouzadeh-Ghadikolai O., von Lewinski D., Rothenhäusler H.B., Theokas S., Robier C., Mangge H., Reicht G., Hlade P., Meinitzer A. (2016). Branched-Chain Amino Acids as New Biomarkers of Major Depression—A Novel Neurobiology of Mood Disorder. PLoS ONE.

[41] Han N.R., Kim H.Y., Kim N.R., Lee W.K., Jeong H., Kim H.M., Jeong H.J. (2018). Leucine and glycine dipeptides of porcine placenta ameliorate physical fatigue through enhancing dopaminergic systems. Mol. Med. Rep..

[42] Solís-Ortiz S., Arriaga-Avila V., Trejo-Bahena A., Guevara-Guzmán R. (2021). Deficiency in the Essential Amino Acids l-Isoleucine, l-Leucine and l-Histidine and Clinical Measures as Predictors of Moderate Depression in Elderly Women: A Discriminant Analysis Study. Nutrients.

[43] Saxena R.N., Pendse V.K., Khanna N.K. (1984). Anti-inflammatory and analgesic properties of four amino-acids. Indian. J. Physiol. Pharmacol..

